# Supplementary material for: Switchable topological polar states in epitaxial BaTiO3 nanoislands on silicon
Source: Nat Commun. 2024 Nov 20;15:10047. doi: 10.1038/s41467-024-54285-z (PMC11579377; doi:10.1038/s41467-024-54285-z)
Supplement: Supplementary file 1 — Supplementary Information [file 41467_2024_54285_MOESM1_ESM.pdf]

Supporting Information

Switchable topological polar states in epitaxial $BaTiO_3$ nanoislands on silicon

Ibukun Olaniyan[1,2]*, Iurii Tikhonov[3], Valentin Hevelke[1,2], Sven Wiesner[1], Leifeng Zhang[4], Anna Razumnaya[5], Nikolay Cherkashin[4], Sylvie Schamm-Chardon[4], Igor Lukyanchuk[3], Dong-Jik Kim[1], Catherine Dubourdieu[1,2]*

[1]Helmholtz-Zentrum Berlin für Materialien und Energie, Hahn-Meitner Platz 1, 14109 Berlin, Germany

[2]Freie Universität Berlin, Physical and Theoretical Chemistry, Arnimallee 22, 14195 Berlin, Germany

[3]Laboratory of Condensed Matter Physics, University of Picardie, 80039, Amiens, France

[4]CEMES-CNRS and Université de Toulouse, 29 rue Jeanne Marvig, 31055 Toulouse, France

[5]Condensed Matter Physics Department, Jožef Stefan Institute, Jamova Cesta 39, 1000, Ljubljana Slovenia

Corresponding authors: catherine.dubourdieu@helmholtz-berlin.de, israel.olaniyan@helmholtz-berlin.de

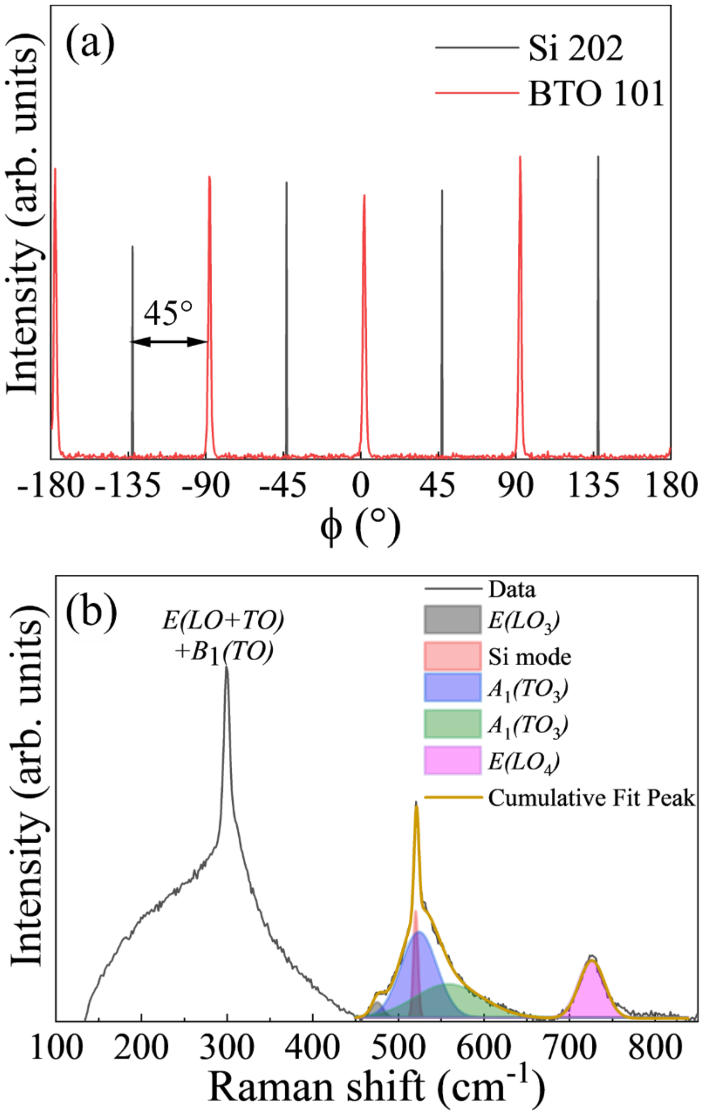

Figure S1: Structural characterization of the $BaTiO_3$ / $SrTiO_3$ / Si heterostructure. (a) Phi scan of the 202 reflection of Si ($\chi = 45°$) and 101 reflection of $BaTiO_3$ ($\chi = 45°$). (b) Raman spectrum of the sample.

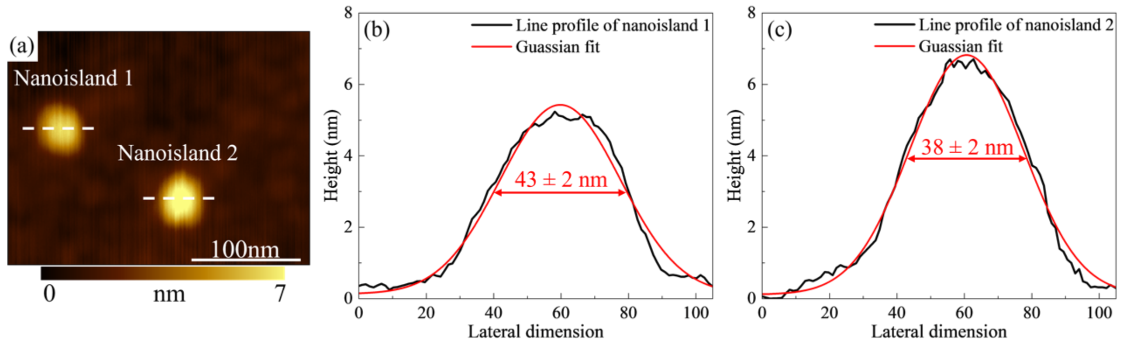

Figure S2: **High resolution AFM image and line scans of nanoislands.** (a) AFM image showing two nanoislands and (b), (c) their line profiles taken along the dashed lines.

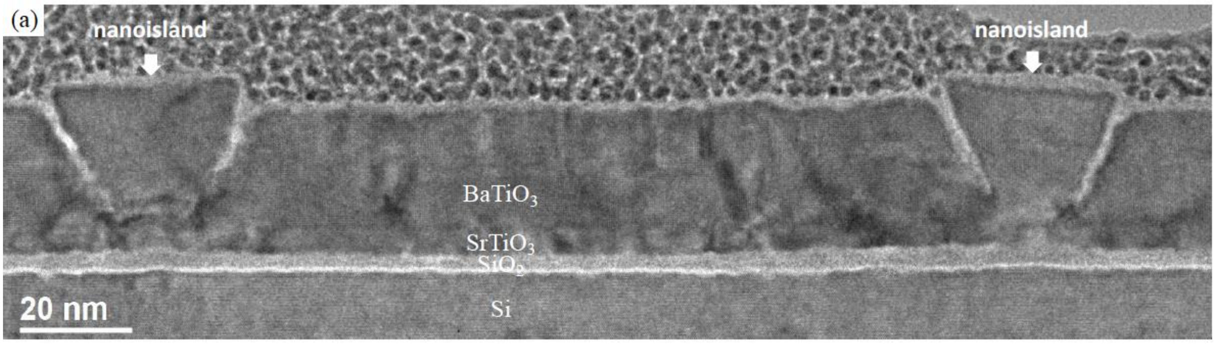

Figure S3: $BaTiO_3$ nanoislands embedded in a $BaTiO_3$ thin film on silicon. Cross-sectional $(110)^{Si}$ TEM image showing two nanoislands in the thin $BaTiO_3$ film epitaxially grown on Si substrate.

(a)

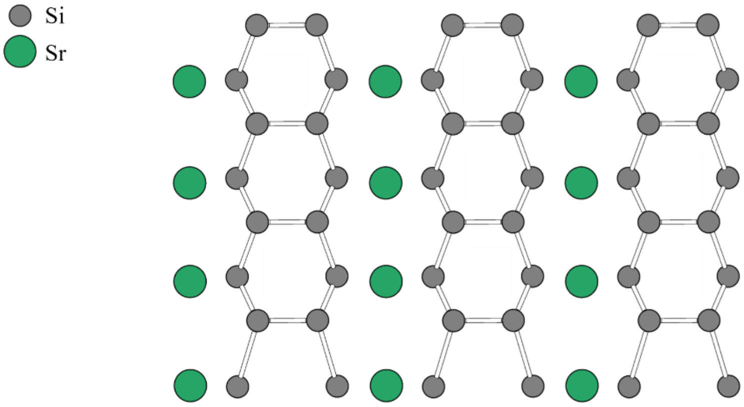

(b)

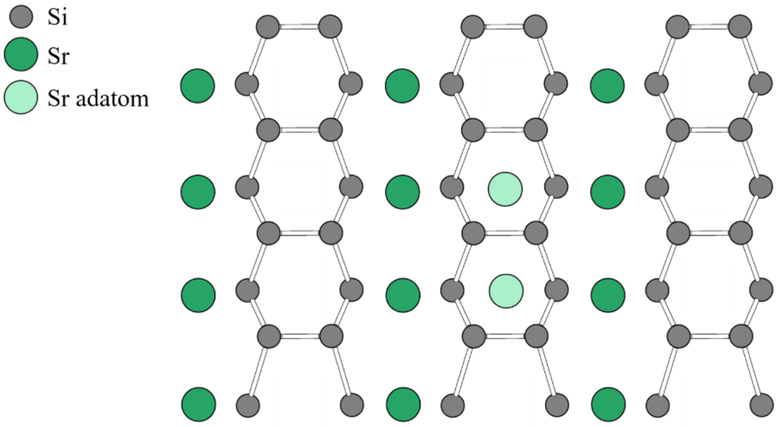

Figure S4: Atomic structure of Sr (2×1) surface reconstruction. (a) Top view of the atomic structure of the Sr (2×1) surface corresponding to ½ ML Sr coverage of the (2×1) dimer-row reconstructed Si surface. (b) Top view of the same surface with two Sr adatoms shown in light green occupying sites on top of a dimer row in between two dimers.

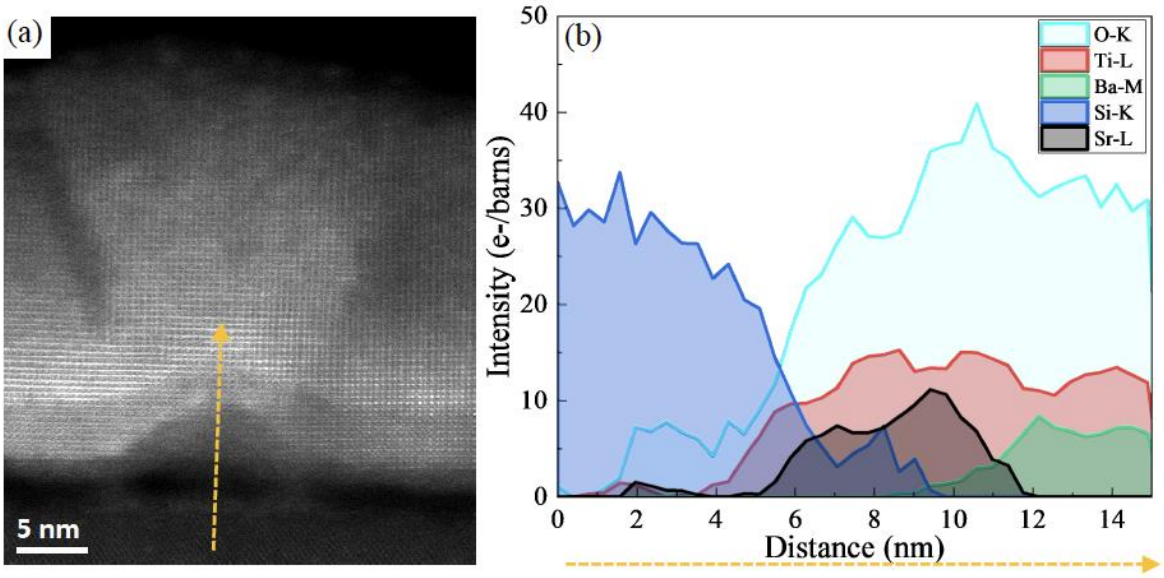

Figure S5: HAADF image and EELS analysis of the bottom of a nanoisland. (a) HAADF image of a nanoisland with a bump clearly seen underneath and (b) elemental profiles of O, Ti, Ba, Sr and Si obtained from acquisition of the corresponding EELS edges (O-K, Ti-L, Ba-M, Sr-L and Si-K) along the orange arrow in (a).

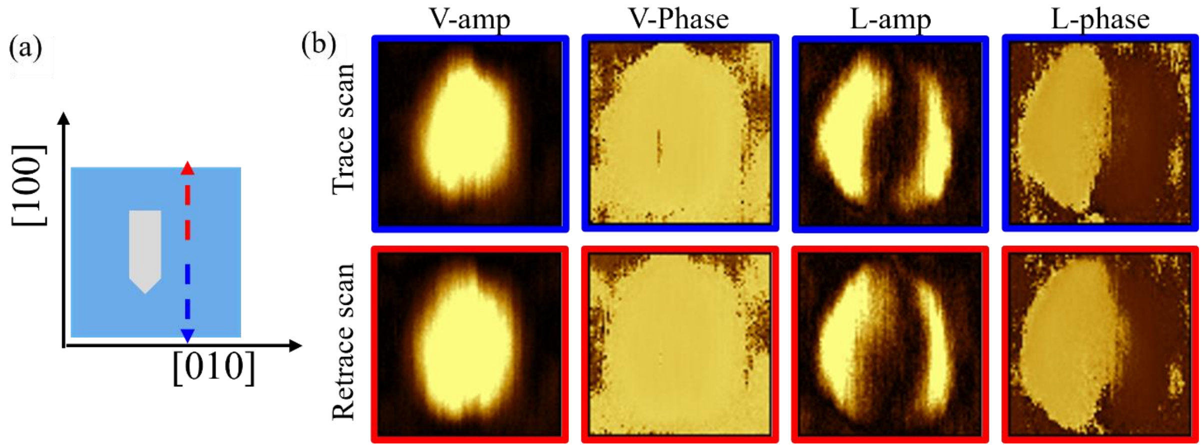

Figure S6: PFM images for trace and retrace scan directions. (a) Schematics showing the trace (red arrow) and retrace (blue arrow) scan directions. (b) PFM images obtained from the trace and retrace scans. The trace images and retrace images are outlined by blue and red rectangles, respectively.

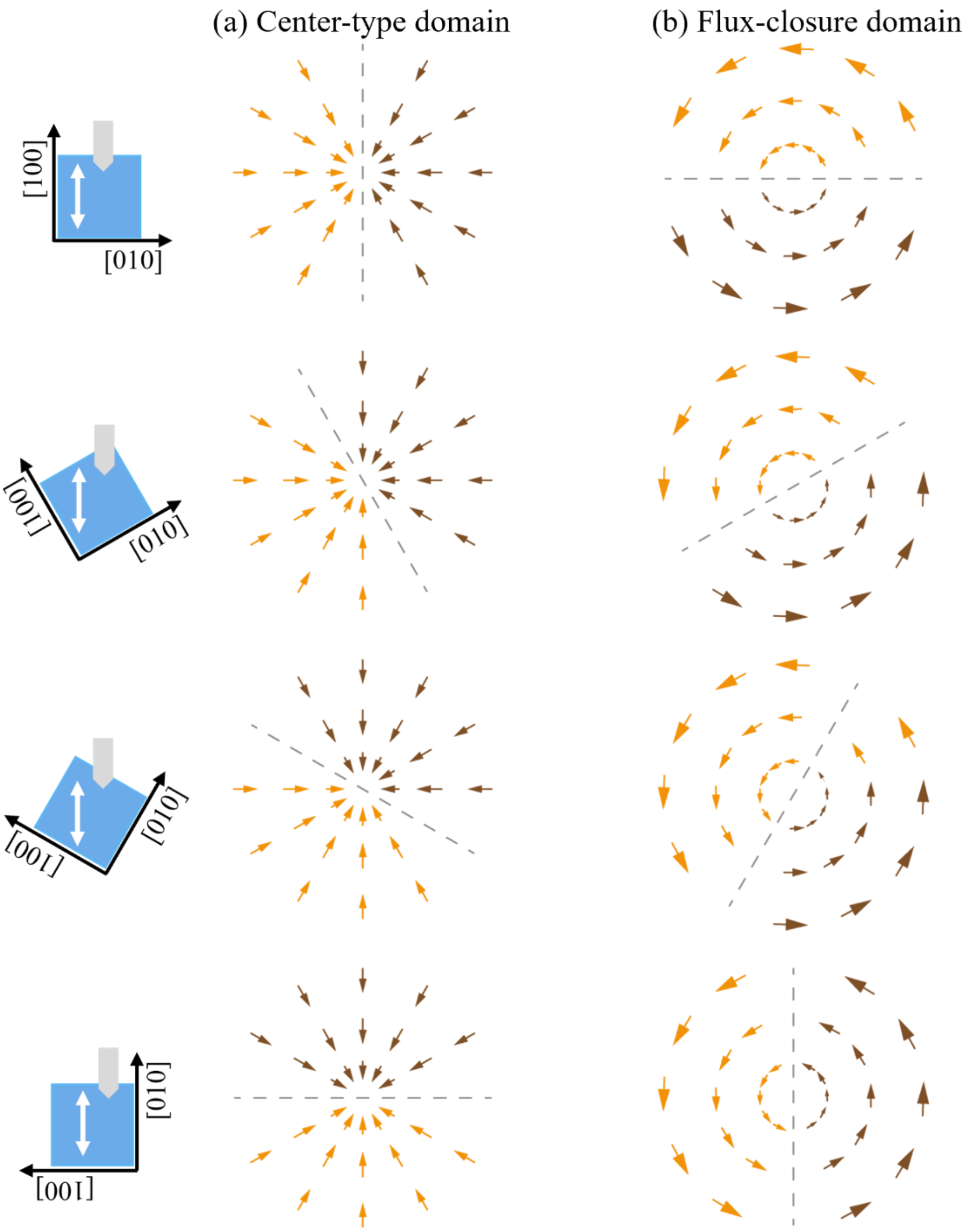

Figure S7: Schematics of center-type and flux-closure patterns with respect to sample rotation. Plan view schematics of (a) center-type domain pattern and (b) flux-closure domain pattern showing the position of domain wall (dashed grey line) as a function of counterclockwise sample rotation. The sample orientation with respect to the cantilever is shown on the left side of the image. The white arrows represent the fast scan direction.

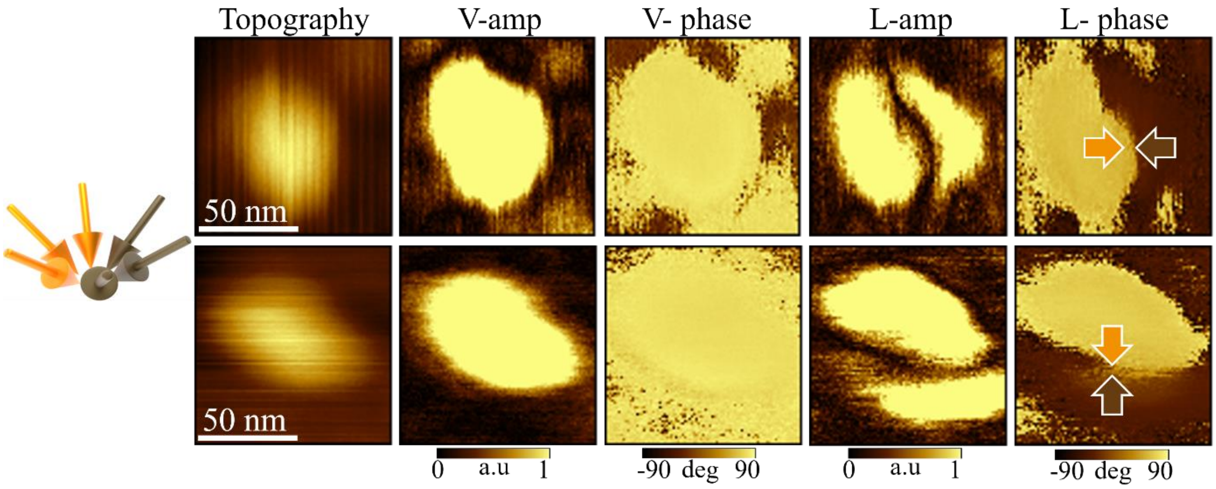

Figure S8: PFM images of a nanoisland at 0° and 90° sample orientation. Amplitude (amp) and phase (phase) images of vertical (V) PFM and lateral (L) PFM captured on a nanoisland at 0° and 90° sample rotation. The left pictogram sketches the central down-converging texture of the polarization vectors.

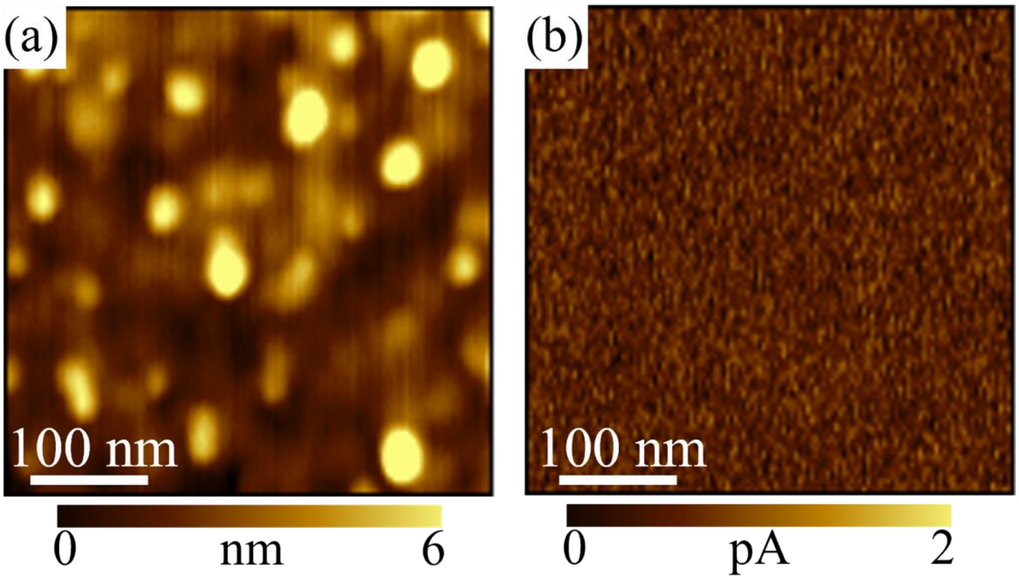

Figure S9: **Topography and corresponding conductive AFM images of nanoislands.** (a) AFM image and (b) the corresponding c-AFM image of the same region, showing the topography and local conductivity, respectively. No contrast in current is observed at the nanoisland locations or at their sidewalls.

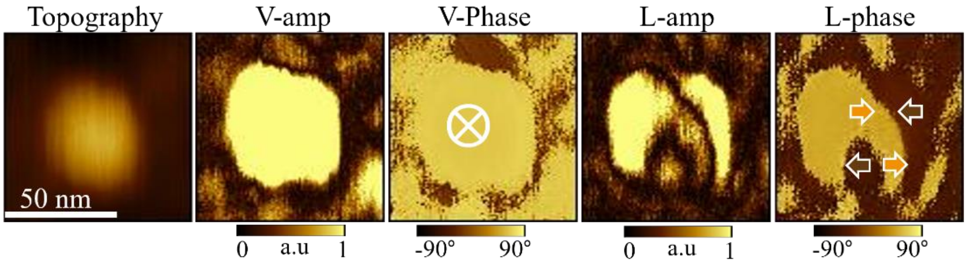

Figure S10: An example of another polarization pattern occasionally observed. Topography, Vertical PFM amplitude and phase, Lateral PFM amplitude and phase images.

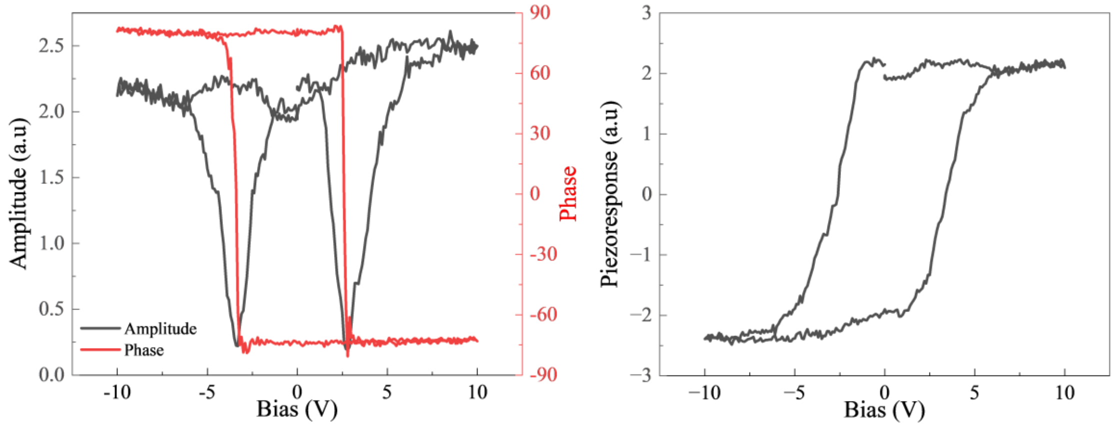

Figure S11: **PFM switching spectroscopy on a nanoisland.** Local PFM switching spectroscopy performed on a nanoisland. Left: PFM amplitude and phase signals (the phase signal sign here is arbitrary) – Right: Piezoresponse signal.
